# Supplementary material for: Spontaneous human CD8 T cell and autoimmune encephalomyelitis-induced CD4/CD8 T cell lesions in the brain and spinal cord of HLA-DRB1*15-positive multiple sclerosis humanized immune system mice
Source: eLife. 2024 Jun 20;12:RP88826. doi: 10.7554/eLife.88826 (PMC11189630; doi:10.7554/eLife.88826)
Supplement: Figure 1—source data 1. [file elife-88826-fig1-data1.docx]

**Figure 1- Source data 1**

| **A ) DR13 MS** | | | | | | | | | | | | | | | | | | | | | | | | | |  |
| --- | --- | --- | --- | --- | --- | --- | --- | --- | --- | --- | --- | --- | --- | --- | --- | --- | --- | --- | --- | --- | --- | --- | --- | --- | --- | --- |
| **Day** | | **hCD45** | | | | | | | | | | | | | | | | | | | | | | | | |
| 7 (non-imm) | | 7,13 | | | | 9,05 | | | 13,40 | | | | 9,74 | | | 11,80 | | | 6,32 | | | 12,00 | | | 6,27 | |
| 13 (non-imm) | | 30,30 | | | | 42,80 | | | 19,40 | | | | 39,00 | | | 40,70 | | | 37,00 | | | 43,10 | | | 45,00 | |
| 42 (non-imm) | | 46,30 | | | | 35,90 | | | 42,80 | | | |  | | |  | | |  | | |  | | |  | |
| 42 spleen (non-imm) | | 52,00 | | | | 84,00 | | | 59,00 | | | |  | | |  | | |  | | |  | | |  | |
|  | |  | | | |  | | |  | | | |  | | |  | | |  | | |  | | |  | |
| 13 (non-imm) | | 30,30 | | | | 42,80 | | | 19,40 | | | | 39,00 | | | 40,70 | | | 37,00 | | | 43,10 | | | 45,00 | |
| 42 (Imm 2x200μg) | | 2,70 | | | | 20,80 | | | 6,12 | | | | 18,40 | | | 4,23 | | |  | | |  | | |  | |
| 42 spleen  (Imm 2x200μg) | | 44,70 | | | | 64,70 | | | 32,70 | | | | 22,90 | | | 41,90 | | |  | | |  | | |  | |
|  | | **mCD45** | | | | | | | | | | | | | | | | | | | | | | | | |
| 7 (non-imm) | | 92,20 | | | | 89,80 | | | 85,90 | | | | 89,50 | | | 87,60 | | | 93,20 | | | 86,90 | | | 93,20 | |
| 13 (non-imm) | | 66,20 | | | | 53,60 | | | 78,30 | | | | 58,50 | | | 56,30 | | | 60,60 | | | 53,70 | | | 52,20 | |
| 42 (non-imm) | | 51,80 | | | | 62,10 | | | 56,10 | | | |  | | |  | | |  | | |  | | |  | |
| 42 spleen (non-imm) | | 46,90 | | | | 12,40 | | | 39,60 | | | |  | | |  | | |  | | |  | | |  | |
|  | |  | | | |  | | |  | | | |  | | |  | | |  | | |  | | |  | |
| 13 (non-imm) | | 66,20 | | | | 53,60 | | | 78,30 | | | | 58,50 | | | 56,30 | | | 60,60 | | | 53,70 | | | 52,20 | |
| 42 (Imm 2x200μg) | | 97,20 | | | | 78,30 | | | 93,40 | | | | 80,10 | | | 95,30 | | |  | | |  | | |  | |
| 42 spleen  (Imm 2x200μg) | | 51,10 | | | | 33,10 | | | 65,60 | | | | 74,90 | | | 52,20 | | |  | | |  | | |  | |
|  | |  | | | |  | | |  | | | |  | | |  | | |  | | |  | | |  | |
| **DR15 HI** |  | |  | |  | | |  | | |  | | |  | | |  | | |  | | |  | | | |
| **Day** | | **hCD45** | | | | | | | | | | | | | | | | | | | | | | | | |
| 7 (non-imm) | | 1,03 | | | | 2,68 | | | 1,44 | | | | 4,70 | | | 7,17 | | | 3,82 | | | 5,94 | | | 5,04 | |
| 13 (non-imm) | | 6,99 | | | | 7,03 | | | 1,43 | | | | 26,30 | | | 29,60 | | | 12,90 | | | 24,40 | | | 16,60 | |
| 42 (non-imm) | | 4,73 | | | | 34,60 | | | 5,12 | | | |  | | |  | | |  | | |  | | |  | |
| 42 spleen (non-imm) | | 31,60 | | | | 54,60 | | | 26,60 | | | |  | | |  | | |  | | |  | | |  | |
|  | |  | | | |  | | |  | | | |  | | |  | | |  | | |  | | |  | |
| 13 (non-imm) | | 6,99 | | | | 7,03 | | | 1,43 | | | | 26,30 | | | 29,60 | | | 12,90 | | | 24,40 | | | 16,60 | |
| 42 (Imm 2x200μg) | | 11,70 | | | | 2,27 | | | 7,30 | | | | 5,13 | | | 26,20 | | |  | | |  | | |  | |
| 42 spleen  (Imm 2x200μg) | | 60,20 | | | | 7,61 | | | 18,60 | | | | 14,40 | | | 33,90 | | |  | | |  | | |  | |
|  | | **mCD45** | | | | | | | | | | | | | | | | | | | | | | | | |
| 7 (non-imm) | | 98,90 | | | | 96,90 | | | 98,20 | | | | 94,80 | | | 91,30 | | | 95,40 | | | 93,60 | | | 94,50 | |
| 13 (non-imm) | | 92,30 | | | | 92,80 | | | 98,10 | | | | 71,60 | | | 68,20 | | | 85,50 | | | 66,90 | | | 81,20 | |
| 42 (non-imm) | | 94,00 | | | | 62,10 | | | 93,40 | | | |  | | |  | | |  | | |  | | |  | |
| 42 spleen (non-imm) | | 68,30 | | | | 45,30 | | | 73,30 | | | |  | | |  | | |  | | |  | | |  | |
|  | |  | | | |  | | |  | | | |  | | |  | | |  | | |  | | |  | |
| 13 (non-imm) | | 92,30 | | | | 92,80 | | | 98,10 | | | | 71,60 | | | 68,20 | | | 85,50 | | | 66,90 | | | 81,20 | |
| 42 (Imm 2x200μg) | | 87,60 | | | | 97,30 | | | 92,10 | | | | 94,50 | | | 73,20 | | |  | | |  | | |  | |
| 42 spleen  (Imm 2x200μg) | | 39,60 | | | | 90,20 | | | 78,30 | | | | 85,00 | | | 65,40 | | |  | | |  | | |  | |
| **DR15 MS1** | | | | | | | | | | | | | | | | | | | | | | | | | | |
| **Day** | | **hCD45** | | | | | | | | | | | | | | | | | | | | | | | | |
| 7 (non-imm) | | 1,69 | | | | 1,08 | | | 2,07 | | | | 2,91 | | | 3,78 | | | 0,68 | | |  | | |  | |
| 13 (non-imm) | | 24,20 | | | | 26,90 | | | 27,90 | | | | 21,40 | | | 31,30 | | | 42,40 | | | 4,76 | | |  | |
| 42 (non-imm) | | 31,80 | | | | 34,90 | | | 37,40 | | | |  | | |  | | |  | | |  | | |  | |
| 42 spleen (non-imm) | | 58,70 | | | | 64,00 | | | 62,90 | | | |  | | |  | | |  | | |  | | |  | |
|  | |  | | | |  | | |  | | | |  | | |  | | |  | | |  | | |  | |
| 13 (non-imm) | | 24,20 | | | | 26,90 | | | 27,90 | | | | 21,40 | | | 31,30 | | | 42,40 | | | 4,76 | | |  | |
| 42 (Imm 2x200μg) | | 12,30 | | | | 21,60 | | | 14,40 | | | | 12,50 | | | 17,50 | | |  | | |  | | |  | |
| 42 spleen  (Imm 2x200μg) | | 45,40 | | | | 39,00 | | | 20,40 | | | | 40,00 | | | 35,20 | | |  | | |  | | |  | |
|  | | **mCD45** | | | | | | | | | | | | | | | | | | | | | | | | |
| 7 (non-imm) | | 97,90 | | | | 98,80 | | | 97,30 | | | | 96,80 | | | 95,60 | | | 99,10 | | |  | | |  | |
| 13 (non-imm) | | 74,50 | | | | 72,40 | | | 71,30 | | | | 77,30 | | | 67,00 | | | 56,10 | | | 95,00 | | |  | |
| 42 (non-imm) | | 67,30 | | | | 64,50 | | | 61,00 | | | |  | | |  | | |  | | |  | | |  | |
| 42 spleen (non-imm) | | 41,20 | | | | 35,90 | | | 37,00 | | | |  | | |  | | |  | | |  | | |  | |
|  | |  | | | |  | | |  | | | |  | | |  | | |  | | |  | | |  | |
| 13 (non-imm) | | 74,50 | | | | 72,40 | | | 71,30 | | | | 77,30 | | | 67,00 | | | 56,10 | | | 95,00 | | |  | |
| 42 (Imm 2x200μg) | | 87,30 | | | | 77,20 | | | 84,90 | | | | 86,80 | | | 81,70 | | |  | | |  | | |  | |
| 42 spleen  (Imm 2x200μg) | | 53,30 | | | | 60,70 | | | 79,40 | | | | 59,80 | | | 64,50 | | |  | | |  | | |  | |
| **DR15 MS2** | | | | | | | | | | | | | | | | | | | | | | | | | | |
| **Day** | | **hCD45** | | | | | | | | | | | | | | | | | | | | | | | | |
| 7 (non-imm) | | 3,73 | | | | 4,39 | | | 3,99 | | | | 4,91 | | | 4,64 | | | 0,53 | | | 1,55 | | |  | |
| 13 (non-imm) | | 22,30 | | | | 22,90 | | | 23,50 | | | | 29,80 | | | 13,30 | | | 3,71 | | | 38,50 | | |  | |
| 42 (non-imm) | | 53,70 | | | | 52,10 | | | 54,50 | | | |  | | |  | | |  | | |  | | |  | |
| 42 spleen (non-imm) | | 66,00 | | | | 48,10 | | | 66,70 | | | |  | | |  | | |  | | |  | | |  | |
|  | |  | | | |  | | |  | | | |  | | |  | | |  | | |  | | |  | |
| 13 (non-imm) | | 22,30 | | | | 22,90 | | | 23,50 | | | | 29,80 | | | 13,30 | | | 3,71 | | | 38,50 | | |  | |
| 42 (Imm 1x100μg) | | 52,10 | | | | 58,60 | | | 47,80 | | | | 23,60 | | |  | | |  | | |  | | |  | |
| 42 spleen  (Imm 1x100μg) | | 54,70 | | | | 73,40 | | | 72,10 | | | | 28,80 | | |  | | |  | | |  | | |  | |
|  | | **mCD45** | | | | | | | | | | | | | | | | | | | | | | | | |
| 7 (non-imm) | | 96,10 | | | | 95,50 | | | 95,90 | | | | 94,9 | | | 95,20 | | | 99,40 | | | 98,2 | | |  | |
| 13 (non-imm) | | 77,30 | | | | 76,50 | | | 76,10 | | | | 69,6 | | | 86,50 | | | 96,10 | | | 61,0 | | |  | |
| 42 (non-imm) | | 45,50 | | | | 46,90 | | | 44,40 | | | |  | | |  | | |  | | |  | | |  | |
| 42 spleen (non-imm) | | 33,30 | | | | 50,50 | | | 31,40 | | | |  | | |  | | |  | | |  | | |  | |
|  | |  | | | |  | | |  | | | |  | | |  | | |  | | |  | | |  | |
| 13 (non-imm) | | 77,30 | | | | 76,50 | | | 76,10 | | | | 69,6 | | | 86,50 | | | 96,10 | | | 61,0 | | |  | |
| 42 (Imm 1x100μg) | | 45,00 | | | | 39,30 | | | 51,20 | | | | 75,7 | | |  | | |  | | |  | | |  | |
| 42 spleen  (Imm 1x100μg) | | 44,70 | | | | 26,00 | | | 26,50 | | | | 70,8 | | |  | | |  | | |  | | |  | |
| **B) DR13 MS** | | | | | | | | | | | | | | | | | | | | | | | | | |  |
| **Day** | | **CD4 T cells** | | | | | | | | | | | | | | | | | | | | | | | |  |
| 7 (non-imm) | | 53,6 | | | | 52,3 | | | 49,9 | | | | 44,6 | | | 53,1 | | | 53,0 | | | 46,6 | | | 53,2 | |
| 13 (non-imm) | | 72,4 | | | | 77,6 | | | 81,7 | | | | 80,4 | | | 75,6 | | | 77,1 | | | 76,2 | | | 83,8 | |
| 42 (non-imm) | | 80,9 | | | | 86,8 | | | 87,0 | | | |  | | |  | | |  | | |  | | |  | |
| 42 (imm 2x200μg) | | 78,8 | | | | 86,7 | | | 62,4 | | | | 90,4 | | | 74,7 | | |  | | |  | | |  | |
|  | | **CD8 T cells** | | | | | | | | | | | | | | | | | | | | | | | |  |
| 7 (non-imm) | | 32,9 | | | | 29,9 | | | 31,9 | | | | 36,5 | | | 30,7 | | | 30,2 | | | 28,8 | | | 31,3 | |
| 13 (non-imm) | | 23,2 | | | | 18,3 | | | 15,5 | | | | 17,1 | | | 20,0 | | | 21,2 | | | 20,9 | | | 13,3 | |
| 42 (non-imm) | | 21,1 | | | | 13,4 | | | 12,4 | | | |  | | |  | | |  | | |  | | |  | |
| 42 (imm 2x200μg) | | 23,1 | | | | 17,9 | | | 44,8 | | | | 12,2 | | | 30,1 | | |  | | |  | | |  | |
| **DR15 HI** | | | | | | | | | | | | | | | | | | | | | | | | | | |
| Day | | **CD4 T cells** | | | | | | | | | | | | | | | | | | | | | | | | |
| 7 (non-imm) | | 76,3 | | | | 61,0 | | | 79,3 | | | | 74,8 | | | 74,9 | | | 68,8 | | | 78,0 | | | 72,7 | |
| 13 (non-imm) | | 65,5 | | | | 70,9 | | | 64,9 | | | | 62,3 | | | 69,1 | | | 58,8 | | | 34,1 | | | 73,1 | |
| 42 (non-imm) | | 62,5 | | | | 90,0 | | | 77,7 | | | |  | | |  | | |  | | |  | | |  | |
| 42 (imm 2x200μg) | | 85,2 | | | | 82,2 | | | 88,2 | | | | 85,7 | | | 87,7 | | |  | | |  | | |  | |
|  | | **CD8 T cells** | | | | | | | | | | | | | | | | | | | | | | | | |
| 7 (non-imm) | | 20,1 | | | | 18,6 | | | 14,6 | | | | 20,9 | | | 19,6 | | | 25,0 | | | 19,5 | | | 27,3 | |
| 13 (non-imm) | | 27,6 | | | | 22,3 | | | 26,0 | | | | 30,0 | | | 23,9 | | | 43,1 | | | 66,7 | | | 26,9 | |
| 42 (non-imm) | | 37,5 | | | | 11,0 | | | 23,2 | | | |  | | |  | | |  | | |  | | |  | |
| 42 (imm 2x200μg) | | 16,0 | | | | 17,8 | | | 15,8 | | | | 18,1 | | | 14,5 | | |  | | |  | | |  | |
| **DR15 MS1** | | | | | | | | | | | | | | | | | | | | | | | | | | |
| **Day** | | **CD4 T cells** | | | | | | | | | | | | | | | | | | | | | | | | |
| 7 (non-imm) | | 73,5 | | | | 62,1 | | | 81,8 | | | | 63,3 | | | 47,1 | | | 58,1 | | |  | | |  | |
| 13 (non-imm) | | 48,4 | | | | 59,3 | | | 54,6 | | | | 67,5 | | | 53,9 | | | 50,1 | | | 66,3 | | |  | |
| 42 (non-imm) | | 54,2 | | | | 68,4 | | | 62,9 | | | |  | | |  | | |  | | |  | | |  | |
| 42 (imm 2x200μg) | | 70,4 | | | | 73,9 | | | 63,7 | | | | 53,3 | | | 76,1 | | |  | | |  | | |  | |
|  | | **CD8 T cells** | | | | | | | | | | | | | | | | | | | | | | | | |
| 7 (non-imm) | | 24,5 | | | | 34,5 | | | 18,2 | | | | 26,7 | | | 41,2 | | | 35,5 | | |  | | |  | |
| 13 (non-imm) | | 53,3 | | | | 43,8 | | | 46,6 | | | | 32,5 | | | 46,4 | | | 50,7 | | | 35,1 | | |  | |
| 42 (non-imm) | | 46,6 | | | | 35,2 | | | 40,3 | | | |  | | |  | | |  | | |  | | |  | |
| 42 (imm 2x200μg) | | 29,2 | | | | 28,8 | | | 35,0 | | | | 43,1 | | | 27,7 | | |  | | |  | | |  | |
| **DR15 MS2** | | | | | | | | | | | | | | | | | | | | | | | | | | |
| Day | | **CD4 T cells** | | | | | | | | | | | | | | | | | | | | | | | | |
| 7 (non-imm) | | 61,5 | | | | 64,2 | | | 65,2 | | | | 68,8 | | | 72,8 | | | 54,5 | | | 59,7 | | |  | |
| 13 (non-imm) | | 67,1 | | | | 64,0 | | | 65,6 | | | | 53,8 | | | 78,5 | | | 62,9 | | | 68,9 | | |  | |
| 42 (non-imm) | | 91,7 | | | | 73,2 | | | 53,4 | | | |  | | |  | | |  | | |  | | |  | |
| 42 (imm 1x100μg) | | 91,9 | | | | 93,0 | | | 85,8 | | | | 74,7 | | |  | | |  | | |  | | |  | |
|  | | **CD8 T cells** | | | | | | | | | | | | | | | | | | | | | | | | |
| 7 (non-imm) | | 32,30 | | | | 28,00 | | | 26,9 | | | | 25,7 | | | 22,1 | | | 38,6 | | | 34,1 | | |  | |
| 13 (non-imm) | | 27,30 | | | | 29,40 | | | 28,5 | | | | 41,0 | | | 18,3 | | | 33,0 | | | 27,1 | | |  | |
| 42 (non-imm) | | 6,65 | | | | 24,90 | | | 44,6 | | | |  | | |  | | |  | | |  | | |  | |
| 42 (imm 1x100μg) | | 6,11 | | | | 4,63 | | | 10,4 | | | | 19,6 | | |  | | |  | | |  | | |  | |
| **C) Spleen dpt42** | | | | | | | | | | | | | | | | | | | | | | | | | | |
| **DR13 MS** | | **CD4 T cells** | | | | | | | | | | | | | | | | | | | | | | | | |
| non-imm | | 43,80 | | 53,10 | | | 54,70 | | |  | |  | | |  | | |  | | |  | | |  | | |
| imm 2x200μg | | 35,20 | | 43,60 | | | 40,50 | | | 60,70 | | 45,10 | | |  | | |  | | |  | | |  | | |
|  | | **CD8 T cells** | | | | | | | | | | | | | | | | | | | | | | | | |
| non-imm | | 23,00 | | 6,88 | | | 17,50 | | |  | |  | | |  | | |  | | |  | | |  | | |
| imm 2x200μg | | 11,00 | | 10,30 | | | 21,80 | | | 8,46 | | 15,90 | | |  | | |  | | |  | | |  | | |
|  | | **CD19 T cells** | | | | | | | | | | | | | | | | | | | | | | | | |
| non-imm | | 27,40 | | 34,60 | | | 23,40 | | |  | |  | | |  | | |  | | |  | | |  | | |
| imm 2x200μg | | 47,00 | | 41,30 | | | 30,30 | | | 23,10 | | 34,00 | | |  | | |  | | |  | | |  | | |
| **DR15 HI** | | **CD4 T cells** | | | | | | | | | | | | | | | | | | | | | | | | |
| non-imm | | 57,00 | | | | 75,50 | | | 60,60 | | | |  | | |  | | |  | | |  | | |  | |
| imm 2x200μg | | 67,80 | | | | 78,30 | | | 64,30 | | | | 72,400 | | | 80,40 | | |  | | |  | | |  | |
|  | | **CD8 T cells** | | | | | | | | | | | | | | | | | | | | | | | | |
| non-imm | | 34,30 | | | | 11,00 | | | 27,90 | | | |  | | |  | | |  | | |  | | |  | |
| imm 2x200μg | | 17,20 | | | | 18,70 | | | 25,20 | | | | 24,30 | | | 16,80 | | |  | | |  | | |  | |
|  | | **CD19 T cells** | | | | | | | | | | | | | | | | | | | | | | | | |
| non-imm | | 2,49 | | | | 2,93 | | | 2,42 | | | |  | | |  | | |  | | |  | | |  | |
| imm 2x200μg | | 2,59 | | | | 1,05 | | | 9,21 | | | | 2,80 | | | 0,51 | | |  | | |  | | |  | |
|  | |  | | | |  | | |  | | | |  | | |  | | |  | | |  | | |  | |
| **DR15 MS1** | | **CD4 T cells** | | | | | | | | | | | | | | | | | | | | | | | | |
| non-imm | | 45,80 | | | | 42,00 | | | 49,40 | | | |  | | |  | | |  | | |  | | |  | |
| imm 2x200μg | | 63,60 | | | | 57,00 | | | 52,90 | | | | 41,50 | | | 62,40 | | |  | | |  | | |  | |
|  | | **CD8 T cells** | | | | | | | | | | | | | | | | | | | | | | | | |
| non-imm | | 49,80 | | | | 43,70 | | | 41,30 | | | |  | | |  | | |  | | |  | | |  | |
| imm 2x200μg | | 33,10 | | | | 36,90 | | | 38,90 | | | | 54,30 | | | 33,30 | | |  | | |  | | |  | |
|  | | **CD19 T cells** | | | | | | | | | | | | | | | | | | | | | | | | |
| non-imm | | 0,54 | | | | 2,99 | | | 1,810 | | | |  | | |  | | |  | | |  | | |  | |
| imm 2x200μg | | 0,67 | | | | 2,03 | | | 1,94 | | | | 1,29 | | | 2,34 | | |  | | |  | | |  | |
|  | |  | | | |  | | |  | | | |  | | |  | | |  | | |  | | |  | |
| **DR15 MS2** | | **CD4 T cells** | | | | | | | | | | | | | | | | | | | | | | | | |
| non-imm | | 82,40 | | | | 55,80 | | | 40,70 | | | |  | | |  | | |  | | |  | | |  | |
| imm 1x100μg | | 88,20 | | | | 85,50 | | | 73,40 | | | | 61,70 | | |  | | |  | | |  | | |  | |
|  | | **CD8 T cells** | | | | | | | | | | | | | | | | | | | | | | | | |
| non-imm | | 9,02 | | | | 39,90 | | | 48,40 | | | |  | | |  | | |  | | |  | | |  | |
| imm 1x100μg | | 8,13 | | | | 7,71 | | | 12,90 | | | | 33,10 | | |  | | |  | | |  | | |  | |
|  | | **CD19 T cells** | | | | | | | | | | | | | | | | | | | | | | | | |
| non-imm | | 7,21 | | | | 1,12 | | | 0,077 | | | |  | | |  | | |  | | |  | | |  | |
| imm 1x100μg | | 1,71 | | | | 5,75 | | | 8,78 | | | | 0,36 | | |  | | |  | | |  | | |  | |
|  | |  | | | |  | | |  | | | |  | | |  | | |  | | |  | | |  | |
| **DR15 MS3** | | **CD4 T cells** | | | | | | | | | | | | | | | | | | | | | | | | |
| non-imm | | 64,10 | | | | 65,70 | | | 59,80 | | | |  | | |  | | |  | | |  | | |  | |
| imm 1x100μg | | 81,60 | | | | 89,40 | | | 76,50 | | | | 86,00 | | |  | | |  | | |  | | |  | |
|  | | **CD8 T cells** | | | | | | | | | | | | | | | | | | | | | | | | |
| non-imm | | 11,80 | | | | 29,20 | | | 12,50 | | | |  | | |  | | |  | | |  | | |  | |
| imm 1x100μg | | 10,70 | | | | 4,22 | | | 15,20 | | | | 9,32 | | |  | | |  | | |  | | |  | |
|  | | **CD19 T cells** | | | | | | | | | | | | | | | | | | | | | | | | |
| non-imm | | 22,20 | | | | 2,37 | | | 21,10 | | | |  | | |  | | |  | | |  | | |  | |
| imm 1x100μg | | 5,44 | | | | 4,71 | | | 4,36 | | | | 1,49 | | |  | | |  | | |  | | |  | |
|  | |  | | | |  | | |  | | | |  | | |  | | |  | | |  | | |  | |
| **DR15 MS4** | | **CD4 T cells** | | | | | | | | | | | | | | | | | | | | | | | | |
| non-imm | | 65,90 | | | | 81,00 | | | 59,80 | | | |  | | |  | | |  | | |  | | |  | |
| imm 1x100μg | | 92,10 | | | | 62,90 | | | 78,90 | | | | 77,40 | | |  | | |  | | |  | | |  | |
|  | | **CD8 T cells** | | | | | | | | | | | | | | | | | | | | | | | | |
| non-imm | | 26,10 | | | | 13,90 | | | 29,400 | | | |  | | |  | | |  | | |  | | |  | |
| imm 1x100μg | | 6,79 | | | | 27,80 | | | 16,500 | | | | 15,70 | | |  | | |  | | |  | | |  | |
|  | | **CD19 T cells** | | | | | | | | | | | | | | | | | | | | | | | | |
| non-imm | | 0,39 | | | | 0,30 | | | 3,07 | | | |  | | |  | | |  | | |  | | |  | |
| imm 1x100μg | | 0,40 | | | | 4,49 | | | 0,36 | | | | 0,20 | | |  | | |  | | |  | | |  | |
|  | |  | | | |  | | |  | | | |  | | |  | | |  | | |  | | |  | |
| **DR15 MS5** | | **CD4 T cells** | | | | | | | | | | | | | | | | | | | | | | | | |
| non-imm | | 74,00 | | | | 75,40 | | | 63,60 | | | |  | | |  | | |  | | |  | | |  | |
| imm 1x100μg | | 68,20 | | | | 70,90 | | | 77,90 | | | | 64,700 | | |  | | |  | | |  | | |  | |
|  | | **CD8 T cells** | | | | | | | | | | | | | | | | | | | | | | | | |
| non-imm | | 16,30 | | | | 15,60 | | | 30,90 | | | |  | | |  | | |  | | |  | | |  | |
| imm 1x100μg | | 25,40 | | | | 18,10 | | | 14,00 | | | | 32,60 | | |  | | |  | | |  | | |  | |
|  | | **CD19 T cells** | | | | | | | | | | | | | | | | | | | | | | | | |
| non-imm | | 7,87 | | | | 7,55 | | | 4,72 | | | |  | | |  | | |  | | |  | | |  | |
| imm 1x100μg | | 4,38 | | | | 7,74 | | | 6,99 | | | | 1,41 | | |  | | |  | | |  | | |  | |
| **D) Splenocytes dpt 42** | | | | | | | | | | | | | | | | | | | | | | | | | | |
| **DR13 MS** | | **CD4^+^IFN-γ^+^** | | | | | | | | | | | | | | | | | | | | | | | | |
| non-imm | | 48,6 | | | | 42,8 | | |  | | | |  | | |  | | |  | | |  | | |  | |
| imm 2x200μg | | 38,7 | | | | 55,7 | | | 55,1 | | | | 61,5 | | | 51,5 | | |  | | |  | | |  | |
|  | | **CD8^+^IFN-γ^+^** | | | | | | | | | | | | | | | | | | | | | | | | |
| non-imm | | 82,7 | | | | 78,0 | | |  | | | |  | | |  | | |  | | |  | | |  | |
| imm 2x200μg | | 62,6 | | | | 82,7 | | | 84,4 | | | | 82,1 | | | 82,2 | | |  | | |  | | |  | |
|  | | **CD4^+^IL-17A^+^** | | | | | | | | | | | | | | | | | | | | | | | | |
| non-imm | | 1,11 | | | | 0,62 | | |  | | | |  | | |  | | |  | | |  | | |  | |
| imm 2x200μg | | 0,72 | | | | 1,07 | | | 2,20 | | | | 2,64 | | | 2,64 | | |  | | |  | | |  | |
|  | |  | | | |  | | |  | | | |  | | |  | | |  | | |  | | |  | |
| **DR15 HI** | | **CD4^+^IFN-γ^+^** | | | | | | | | | | | | | | | | | | | | | | | | |
| non-imm | | 42,5 | | | | 41,6 | | | 47,5 | | | |  | | |  | | |  | | |  | | |  | |
|  | | **CD8^+^IFN-γ^+^** | | | | | | | | | | | | | | | | | | | | | | | | |
| non-imm | | 80,5 | | | |  | | |  | | | |  | | |  | | |  | | |  | | |  | |
|  | | **CD4^+^IL-17A^+^** | | | | | | | | | | | | | | | | | | | | | | | | |
| non-imm | | 0,57 | | | | 0,93 | | | 2,08 | | | |  | | |  | | |  | | |  | | |  | |
|  | |  | | | |  | | |  | | | |  | | |  | | |  | | |  | | |  | |
| **DR15 MS1** | | **CD4^+^IFN-γ^+^** | | | | | | | | | | | | | | | | | | | | | | | | |
| non-imm | | 33,0 | | | | 42,6 | | | 29,5 | | | |  | | |  | | |  | | |  | | |  | |
| imm 2x200μg | | 36,2 | | | | 42,0 | | | 43,4 | | | | 38,5 | | | 44,3 | | |  | | |  | | |  | |
|  | | **CD8^+^IFN-γ^+^** | | | | | | | | | | | | | | | | | | | | | | | | |
| non-imm | | 79,2 | | | | 67,6 | | | 66,1 | | | |  | | |  | | |  | | |  | | |  | |
| imm 2x200μg | | 54,2 | | | | 60,7 | | | 63,8 | | | | 66,7 | | | 63,3 | | |  | | |  | | |  | |
|  | | **CD4^+^IL-17A^+^** | | | | | | | | | | | | | | | | | | | | | | | | |
| non-imm | | 0,92 | | | | 1,49 | | | 2,23 | | | |  | | |  | | |  | | |  | | |  | |
| imm 2x200μg | | 1,09 | | | | 1,39 | | | 1,15 | | | | 0,72 | | | 2,48 | | |  | | |  | | |  | |
|  | |  | | | |  | | |  | | | |  | | |  | | |  | | |  | | |  | |
| **DR15 MS2** | | **CD4^+^IFN-γ^+^** | | | | | | | | | | | | | | | | | | | | | | | | |
| non-imm | | 4,84 | | | | 7,52 | | | 5,02 | | | |  | | |  | | |  | | |  | | |  | |
| imm 1x100μg | | 6,4 | | | | 5,96 | | | 12,3 | | | | 13,2 | | |  | | |  | | |  | | |  | |
|  | | **CD4^-^IFN-γ^+^** | | | | | | | | | | | | | | | | | | | | | | | | |
| non-imm | | 37,40 | | | | 48,20 | | | 63,30 | | | |  | | |  | | |  | | |  | | |  | |
| imm 1x100μg | | 46,6 | | | | 39,30 | | | 55,4 | | | | 63,9 | | |  | | |  | | |  | | |  | |
|  | | **CD4^+^IL-17A^+^** | | | | | | | | | | | | | | | | | | | | | | | | |
| non-imm | | 2,07 | | | | 0,71 | | | 0,240 | | | |  | | |  | | |  | | |  | | |  | |
| imm 1x100μg | | 1,07 | | | | 1,33 | | | 4,10 | | | | 1,15 | | |  | | |  | | |  | | |  | |
|  | | **CD4^-^IL-17A^+^** | | | | | | | | | | | | | | | | | | | | | | | | |
| non-imm | | 1,05 | | | | 0,38 | | | 0,057 | | | |  | | |  | | |  | | |  | | |  | |
| imm 1x100μg | | 0,54 | | | | 0,61 | | | 3,88 | | | | 0,23 | | |  | | |  | | |  | | |  | |
|  | |  | | | |  | | |  | | | |  | | |  | | |  | | |  | | |  | |
| **DR15 MS3** | | **CD4^+^IFN-γ^+^** | | | | | | | | | | | | | | | | | | | | | | | | |
| non-imm | | 11,8 | | | | 9,02 | | | 8,54 | | | |  | | |  | | |  | | |  | | |  | |
| imm 1x100μg | | 6,43 | | | | 6,26 | | | 9,28 | | | | 11,2 | | |  | | |  | | |  | | |  | |
|  | | **CD4^-^IFN-γ^+^** | | | | | | | | | | | | | | | | | | | | | | | | |
| non-imm | | 40,7 | | | | 46,30 | | | 33,80 | | | |  | | |  | | |  | | |  | | |  | |
| imm 1x100μg | | 40,50 | | | | 20,30 | | | 44,90 | | | | 42,1 | | |  | | |  | | |  | | |  | |
|  | | **CD4^+^IL-17A^+^** | | | | | | | | | | | | | | | | | | | | | | | | |
| non-imm | | 2,42 | | | | 2,53 | | | 3,29 | | | |  | | |  | | |  | | |  | | |  | |
| imm 1x100μg | | 4,27 | | | | 4,70 | | | 3,27 | | | | 2,68 | | |  | | |  | | |  | | |  | |
|  | | **CD4^-^IL-17A^+^** | | | | | | | | | | | | | | | | | | | | | | | | |
| non-imm | | 1,80 | | | | 1,06 | | | 2,80 | | | |  | | |  | | |  | | |  | | |  | |
| imm 1x100μg | | 3,17 | | | | 1,75 | | | 1,74 | | | | 0,67 | | |  | | |  | | |  | | |  | |
|  | |  | | | |  | | |  | | | |  | | |  | | |  | | |  | | |  | |
| **DR15 MS5** | | **CD4^+^IFN-γ^+^** | | | | | | | | | | | | | | | | | | | | | | | | |
| non-imm | | 7,83 | | | | 6,72 | | | 9,79 | | | |  | | |  | | |  | | |  | | |  | |
| imm 1x100μg | | 6,11 | | | | 9,52 | | | 4,12 | | | | 3,15 | | |  | | |  | | |  | | |  | |
|  | | **CD4^-^IFN-γ^+^** | | | | | | | | | | | | | | | | | | | | | | | | |
| non-imm | | 31,10 | | | | 33,60 | | | 39,40 | | | |  | | |  | | |  | | |  | | |  | |
| imm 1x100μg | | 22,40 | | | | 28,30 | | | 25,50 | | | | 23,20 | | |  | | |  | | |  | | |  | |
|  | | **CD4^+^IL-17A^+^** | | | | | | | | | | | | | | | | | | | | | | | | |
| non-imm | | 2,87 | | | | 2,58 | | | 2,22 | | | |  | | |  | | |  | | |  | | |  | |
| imm 1x100μg | | 4,79 | | | | 4,32 | | | 2,55 | | | | 1,14 | | |  | | |  | | |  | | |  | |
|  | | **CD4^-^IL-17A^+^** | | | | | | | | | | | | | | | | | | | | | | | | |
| non-imm | | 2,02 | | | | 2,44 | | | 1,62 | | | |  | | |  | | |  | | |  | | |  | |
| imm 1x100μg | | 3,57 | | | | 3,99 | | | 2,73 | | | | 0,76 | | |  | | |  | | |  | | |  | |
|  | |  | | | |  | | |  | | | |  | | |  | | |  | | |  | | |  | |
| 1. **DR13 MS Cell division index** | | | | | | | | | | | | | | | | | | | | | | | | | | |
|  | | non-imm | | | |  | | |  | | | |  | | |  | | |  | | |  | | |  | |
| mMOG35-55 | | 1,114393 | | | | 1,432471 | | | 0,817296 | | | |  | | |  | | |  | | |  | | |  | |
| hMOG35-55 | | 1,285637 | | | | 1,574168 | | | 0,789472 | | | |  | | |  | | |  | | |  | | |  | |
| MOG1-20 | | 1,433565 | | | | 1,568586 | | | 0,907848 | | | |  | | |  | | |  | | |  | | |  | |
| MBP83-99 | | 1,449921 | | | | 1,495482 | | | 1,004507 | | | |  | | |  | | |  | | |  | | |  | |
| anti-CD3 | | 1,530000 | | | | 1,577814 | | | 0,983139 | | | |  | | |  | | |  | | |  | | |  | |
| US | | 1,000000 | | | | 1,000000 | | | 1,000000 | | | |  | | |  | | |  | | |  | | |  | |
|  | | imm 2x200μg | | | |  | | |  | | | |  | | |  | | |  | | |  | | |  | |
| mMOG35-55 | | 3,291701 | | | | 2,614570 | | | 2,089191 | | | | 1,520347 | | |  | | |  | | |  | | |  | |
| hMOG35-55 | | 4,245683 | | | | 2,897676 | | | 2,310165 | | | |  | | |  | | |  | | |  | | |  | |
| MOG1-20 | | 3,672333 | | | | 2,858430 | | | 1,316359 | | | | 1,751039 | | |  | | |  | | |  | | |  | |
| MBP83-99 | | 3,872877 | | | | 3,554057 | | | 1,499571 | | | | 1,931515 | | |  | | |  | | |  | | |  | |
| anti-CD3 | | 4,623396 | | | | 3,362379 | | | 2,180264 | | | |  | | |  | | |  | | |  | | |  | |
| US | | 1,000000 | | | | 1,000000 | | | 1,000000 | | | | 1,000000 | | |  | | |  | | |  | | |  | |
