# Supplementary material for: Spontaneous human CD8 T cell and autoimmune encephalomyelitis-induced CD4/CD8 T cell lesions in the brain and spinal cord of HLA-DRB1*15-positive multiple sclerosis humanized immune system mice
Source: eLife. 2024 Jun 20;12:RP88826. doi: 10.7554/eLife.88826 (PMC11189630; doi:10.7554/eLife.88826)
Supplement: Figure 1—figure supplement 3—source data 1. [file elife-88826-fig1-figsupp3-data1.docx]

**Fig. 1- figure supplement 3A- source data 1: Clinical scores from the EAE tests in C57BL/6 mice.**

EAE clinical scores induced by immunization with myelin peptide antigens in C57/BL6 mice. Groups of mice were immunized with different peptide mixes and amounts as shown in Figure 1- figure supplement 3B.

| **37μg MOG RAT** | dpi12 | dpi13 | dpi14 | dpi15 | dpi16 | dpi17 | dpi18 | dpi19 | dpi20 | dpi21 | dpi22 | dpi23 | dpi24 | dpi25 | dpi26 | dpi27 | dpi28 | dpi29 | dpi30 | dpi31 | dpi32 | dpi33 | dpi35 | dpi36 |
| --- | --- | --- | --- | --- | --- | --- | --- | --- | --- | --- | --- | --- | --- | --- | --- | --- | --- | --- | --- | --- | --- | --- | --- | --- |
| 2 uncut | 0 | 0 | 1 | 2,5 | 3 | 3,5 | 3 | 3 | 3 | 3 | 2,5 | 3 | 3 | 3,5 | 3 | 3 | 3 | 3 | 3 | 3 | 3 | 3 | 3 | 3 |
| 1 1ur | 0,5 | 2 | 3 | 3 | 3 | 3 | 3 | 3,5 | 3 | 3 | 3 | 3 | 2,5 | 2,5 | 2,5 | 2 | 2 | 2 | 2 | 2 | 2 | 2 |  |  |
|  |  |  |  |  |  |  |  |  |  |  |  |  |  |  |  |  |  |  |  |  |  |  |  |  |
| **200μg MOG RAT** |  |  |  |  |  |  |  |  |  |  |  |  |  |  |  |  |  |  |  |  |  |  |  |  |
| 3 1ur | 0 | 0 | 0 | 0,5 | 0,5 | 1,5 | 1,5 | 1 | 1,5 | 1,5 | 1,5 | 1,5 | 1 | 1 | 1 | 1 | 1 | 0,5 | 1 | 1 | 1 | 1 | 1 | 1 |
| 4 uncut | 0 | 0 | 0 | 0,5 | 0,5 | 1,5 | 1,5 | 1,5 | 1 | 1 | 1 | 1 | 1 | 0,5 | 0,5 | 0,5 | 0,5 | 0,5 | 1 | 1 | 1 | 1 | 1,5 | 1,5 |
|  |  |  |  |  |  |  |  |  |  |  |  |  |  |  |  |  |  |  |  |  |  |  |  |  |
| **200μg of all peptides** |  |  |  |  |  |  |  |  |  |  |  |  |  |  |  |  |  |  |  |  |  |  |  |  |
| 5 1ll | 0 | 0 | 0 | 0 | 0 | 0 | 0 | 0 | 2 | 3 | 3 | 3 | 3 | 3 | 3 | 3 | 3 | 3 | 3 | 2,5 | 2,5 | 2,5 | 2,5 | 2,5 |
| 6 uncut | 0 | 0 | 0 | 0 | 0 | 0 | 0 | 0 | 0 | 0 | 0,5 | 1,5 | 2 | 2 | 2 | 2 | 2 | 2 | 2 | 1,5 | 1,5 | 1 | 0,5 | 0,5 |
