# Supplementary material for: Spontaneous human CD8 T cell and autoimmune encephalomyelitis-induced CD4/CD8 T cell lesions in the brain and spinal cord of HLA-DRB1*15-positive multiple sclerosis humanized immune system mice
Source: eLife. 2024 Jun 20;12:RP88826. doi: 10.7554/eLife.88826 (PMC11189630; doi:10.7554/eLife.88826)
Supplement: Figure 1—figure supplement 4—source data 1. [file elife-88826-fig1-figsupp4-data1.docx]

**Fig. 1- figure supplement 4- source data 1: Reconstitution of a human adaptive immune system in B2m-NOG mice engrafted with PBMC from MS patients.**

1. DR15 MS3

| Day | hCD45 | | | | | | |
| --- | --- | --- | --- | --- | --- | --- | --- |
| 7 (non-imm) | 5,35 | 4,49 | 5,3 | 2,35 | 2,68 | 3,52 | 2,15 |
| 13 (non-imm) | 54,50 | 35,40 | 37,3 | 51,50 | 34,80 | 53,30 | 47,80 |
| 42 (non-imm) | 84,70 | 41,90 | 83,7 |  |  |  |  |
| 42 spleen (non-imm) | 88,00 | 51,40 | 81,9 |  |  |  |  |
|  |  |  |  |  |  |  |  |
| 13 (non-imm) | 54,50 | 35,40 | 37,3 | 51,50 | 34,80 | 53,30 | 47,80 |
| 42 (Imm 1x100μg) | 72,60 | 72,80 | 57,5 | 46,90 |  |  |  |
| 42 spleen  (Imm 1x100μg) | 77,70 | 67,30 | 63,9 | 51,00 |  |  |  |
|  | mCD45 | | | | | | |
| 7 (non-imm) | 94,60 | 95,40 | 94,50 | 97,5 | 97,3 | 96,4 | 97,8 |
| 13 (non-imm) | 44,30 | 63,30 | 61,70 | 46,4 | 63,8 | 45,6 | 49,8 |
| 42 (non-imm) | 15,30 | 58,00 | 16,20 |  |  |  |  |
| 42 spleen (non-imm) | 11,80 | 47,90 | 17,10 |  |  |  |  |
|  |  |  |  |  |  |  |  |
| 13 (non-imm) | 44,30 | 63,30 | 61,70 | 46,4 | 63,8 | 45,6 | 49,8 |
| 42 (Imm 1x100μg) | 27,30 | 27,20 | 42,30 | 53,0 |  |  |  |
| 42 spleen  (Imm 1x100μg) | 21,90 | 32,20 | 35,90 | 48,6 |  |  |  |

DR15 MS4

| Day | hCD45 | | | | | | |
| --- | --- | --- | --- | --- | --- | --- | --- |
| 7 (non-imm) | 1,87 | 3,02 | 4,51 | 6,50 | 2,71 | 2,17 | 4,48 |
| 13 (non-imm) | 26,40 | 27,40 | 26,70 | 22,90 | 17,20 | 28,20 | 28,50 |
| 42 (non-imm) | 25,40 | 5,77 |  |  |  |  |  |
| 42 spleen (non-imm) | 14,90 | 27,00 | 5,97 |  |  |  |  |
|  |  |  |  |  |  |  |  |
| 13 (non-imm) | 26,40 | 27,40 | 26,70 | 22,90 | 17,20 | 28,20 | 28,50 |
| 42 (Imm 1x100μg) | 38,80 | 9,56 | 12,50 | 28,90 |  |  |  |
| 42 spleen  (Imm 1x100μg) | 39,80 | 9,95 | 20,10 | 12,50 |  |  |  |
|  | mCD45 | | | | | | |
| 7 (non-imm) | 97,90 | 96,60 | 95,20 | 93,0 | 97,0 | 97,6 | 94,8 |
| 13 (non-imm) | 73,00 | 72,30 | 73,10 | 76,7 | 82,6 | 71,3 | 71,2 |
| 42 (non-imm) | 74,50 | 94,20 |  |  |  |  |  |
| 42 spleen (non-imm) | 78,10 | 71,90 | 93,50 |  |  |  |  |
|  |  |  |  |  |  |  |  |
| 13 (non-imm) | 73,00 | 72,30 | 73,10 | 76,7 | 82,6 | 71,3 | 71,2 |
| 42 (Imm 1x100μg) | 61,00 | 90,30 | 87,50 | 70,7 |  |  |  |
| 42 spleen  (Imm 1x100μg) | 59,80 | 88,80 | 78,60 | 86,2 |  |  |  |

DR15 MS5

| Day | hCD45 | | | | | | |
| --- | --- | --- | --- | --- | --- | --- | --- |
| 7 (non-imm) | 10,3 | 7,27 | 6,37 | 3,81 | 3,78 | 3,67 | 2,87 |
| 13 (non-imm) | 23,7 | 39,20 | 30,00 | 46,20 | 32,90 | 45,10 | 32,90 |
| 42 (non-imm) | 76,3 | 77,30 | 87,20 |  |  |  |  |
| 42 spleen (non-imm) | 75,9 | 67,50 | 85,60 |  |  |  |  |
|  |  |  |  |  |  |  |  |
| 13 (non-imm) | 23,7 | 39,20 | 30,00 | 46,20 | 32,90 | 45,10 | 32,90 |
| 42 (Imm 1x100μg) | 47,5 | 73,30 | 83,90 | 83,70 |  |  |  |
| 42 spleen  (Imm 1x100μg) | 45,4 | 79,30 | 80,90 | 78,70 |  |  |  |
|  | mCD45 | | | | | | |
| 7 (non-imm) | 89,1 | 92,4 | 93,2 | 95,9 | 95,9 | 95,6 | 96,8 |
| 13 (non-imm) | 75,5 | 60,4 | 69,5 | 52,9 | 66,1 | 53,7 | 66,7 |
| 42 (non-imm) | 23,7 | 22,5 | 12,7 |  |  |  |  |
| 42 spleen (non-imm) | 23,5 | 31,6 | 13,7 |  |  |  |  |
|  |  |  |  |  |  |  |  |
| 13 (non-imm) | 75,5 | 60,4 | 69,5 | 52,9 | 66,1 | 53,7 | 66,7 |
| 42 (Imm 1x100μg) | 52,4 | 26,6 | 16,1 | 16,1 |  |  |  |
| 42 spleen  (Imm 1x100μg) | 53,4 | 19,8 | 18,8 | 20,8 |  |  |  |

1. DR15 MS3

| Day | CD4 T cells | | | | | | |
| --- | --- | --- | --- | --- | --- | --- | --- |
| 7 (non-imm) | 69,7 | 65,0 | 65,8 | 63,6 | 66,1 | 64,6 | 67,7 |
| 13 (non-imm) | 74,0 | 61,3 | 71,6 | 67,5 | 77,2 | 76,6 | 77,0 |
| 42 (non-imm) | 84,6 | 72,9 | 88,1 |  |  |  |  |
| 42 (imm 1x100μg) | 91,9 | 95,7 | 83,2 | 91,4 |  |  |  |
|  | CD8 T cells | | | | | | |
| 7 (non-imm) | 27,90 | 30,80 | 30,2 | 32,80 | 29,0 | 30,0 | 28,2 |
| 13 (non-imm) | 20,90 | 25,20 | 23,7 | 25,50 | 18,1 | 18,4 | 18,5 |
| 42 (non-imm) | 14,20 | 25,90 | 11,0 |  |  |  |  |
| 42 (imm 1x100μg) | 7,47 | 3,96 | 14,8 | 7,36 |  |  |  |

DR15 MS4

| Day | CD4 T cells | | | | | | |
| --- | --- | --- | --- | --- | --- | --- | --- |
| 7 (non-imm) | 72,4 | 68,3 | 74,0 | 70,8 | 65,9 | 68,4 | 58,7 |
| 13 (non-imm) | 69,7 | 67,6 | 56,3 | 52,9 | 56,9 | 54,4 | 53,1 |
| 42 (non-imm) | 57,5 | 92,3 | 74,8 |  |  |  |  |
| 42 (imm 1x100μg) | 95,1 | 77,1 | 88,7 | 89,2 |  |  |  |
|  | CD8 T cells | | | | | | |
| 7 (non-imm) | 23,10 | 24,60 | 21,20 | 22,70 | 31,0 | 26,9 | 34,9 |
| 13 (non-imm) | 23,00 | 25,90 | 35,10 | 39,60 | 36,6 | 41,2 | 38,8 |
| 42 (non-imm) | 38,10 | 6,84 | 21,40 |  |  |  |  |
| 42 (imm 1x100μg) | 4,49 | 19,00 | 9,69 | 9,19 |  |  |  |

DR15 MS5

| Day | CD4 T cells | | | | | | |
| --- | --- | --- | --- | --- | --- | --- | --- |
| 7 (non-imm) | 81,10 | 83,80 | 81,80 | 80,40 | 83,40 | 85,30 | 85,50 |
| 13 (non-imm) | 67,80 | 66,60 | 67,40 | 67,30 | 60,80 | 71,50 | 74,20 |
| 42 (non-imm) | 81,70 | 86,80 | 69,80 |  |  |  |  |
| 42 (imm 1x100μg) | 79,10 | 87,20 | 85,70 | 72,20 |  |  |  |
|  | CD8 T cells | | | | | | |
| 7 (non-imm) | 13,80 | 12,50 | 14,30 | 15,00 | 11,10 | 8,93 | 9,760 |
| 13 (non-imm) | 27,20 | 27,70 | 27,80 | 29,20 | 33,20 | 23,90 | 21,700 |
| 42 (non-imm) | 17,20 | 12,50 | 29,30 |  |  |  |  |
| 42 (imm 1x100μg) | 20,10 | 12,10 | 13,60 | 27,40 |  |  |  |

1. CFSE low Splenocytes

| DR13 MS | IMM | US | PHA | anti-CD3 | mMOG35-55 | hMOG35-55 | MOG1-20 | MBP83-99 |
| --- | --- | --- | --- | --- | --- | --- | --- | --- |
|  | - | 9,97 | - | 15,23 | 11,13 | 12,83 | 14,3 | 14,47 |
|  |  | 10,37 |  | 16,1 | 14,6 | 16,3 | 16 | 15,25 |
|  |  | 12,4 |  | 12,1 | 10,15 | 9,72 | 11,1 | 12,43 |
|  | + | 2,74 | - | 12,63 | 9,02 | 11,58 | 10,08 | 10,6 |
|  |  | 1,70 |  | 5,47 | 4,43 | 4,92 | 5,03 | 6 |
|  |  | 8,01 |  | 4,20 | 16,73 | 4,68 | 10,46 | 12,03 |
|  |  | 2,02 |  |  | 3,06 |  | 3,54 | 3,92 |
| DR15 HI |  |  |  |  |  |  |  |  |
|  | - | 1,69 | - | 2,27 | 2,18 | 2,51 | 2,54 | 2,52 |
|  |  | 3,40 |  | 4,52 | 4,08 | 4,44 | 4,58 | 4,50 |
|  |  | 0,70 |  | 1,22 | 1,03 | 1,01 | 1,26 | 1,24 |
|  | + | 11,17 | - | 11,9 | 10,96 | 10,86 | 11,93 | 11,65 |
|  |  | 7,01 |  | 8,16 | 6,48 | 8,53 | 7,22 | 9,62 |
|  |  | 4,51 |  | 6,40 | 3,43 | 3,54 | 4,06 | 4,28 |
| DR15 MS1 |  |  |  |  |  |  |  |  |
|  | - | 1,29 | - | 0,82 | 1,06 | 1,24 | 1,35 | 1,32 |
|  |  | 2,91 |  | 2,85 | 2,79 | 3,12 | 3,16 | 3,25 |
|  |  | 5,68 |  | 5,95 | 5,29 | 5,58 | 5,89 | 5,56 |
|  | + | 3,07 | - | 4,29 | 4,28 | 4,85 | 3,58 | 4,48 |
|  |  | 1,60 |  | 1,39 | 1,15 | 1,86 | 1,67 | 1,82 |
|  |  | 3,71 |  | 3,67 | 2,18 | 4,35 |  | 2,86 |
|  |  | 3,69 |  | 3,71 | 4,13 | 3,62 | 4,05 | 3,86 |
|  |  | 6,03 |  | 7,01 | 6,68 | 7,17 | 7,53 | 7,33 |
| DR15 MS2 |  |  |  |  |  |  |  |  |
|  | - | 0,24 | 0,145 | 0,31 | 0,25 | 0,66 | 0,225 | 0,24 |
|  |  | 0,024 | 0,11 | 0,065 | 0,054 | 0,087 | 0,029 | 0,047 |
|  |  | 0,31 | 0,195 | 0,27 | 0,283 | 0,26 | 0,317 | 0,25 |
|  | + | 0,064 | 0,088 | 0,083 | 0,115 | 0,12 | 0,085 | 0,096 |
|  |  | 0,32 | 0,285 | 0,375 | 0,357 | 0,343 | 0,307 | 0,43 |
|  |  | 0,203 | 0,195 | 0,16 | 0,18 | 0,18 | 0,183 | 0,223 |
|  |  | 0,05 | 0,067 | 0,075 | 0,043 | 0,057 | 0,043 | 0,047 |
| DR15 MS3 |  |  |  |  |  |  |  |  |
|  | - | 0,019 | 0,050 | 0,022 | 0,022 | 0,032 | 0,020 | 0,022 |
|  |  | 0,031 | 0,023 | 0,104 | 0,031 | 0,041 | 0,057 | 0,039 |
|  |  | 0,103 | 0,115 | 0,21 | 0,197 | 0,143 | 0,109 | 0,137 |
|  | + | 0,193 | 0,17 | 0,26 | 0,277 | 0,213 | 0,213 | 0,28 |
|  |  | 0,68 | 1,54 | 1,725 | 1,077 | 0,993 | 1,137 | 1,22 |
|  |  | 0,050 | 0,032 | 0,069 | 0,367 | 0,073 | 0,051 | 0,062 |
|  |  | 0,027 | 0,036 | 0,037 | 0,048 | 0,032 | 0,031 | 0,044 |
| DR15 MS4 |  |  |  |  |  |  |  |  |
|  | - | 0,227 | 0,225 | 0,275 | 0,327 | 0,273 | 0,247 | 0,323 |
|  |  | 0,16 | 0,19 | 0,13 | 0,117 | 0,109 | 0,13 | 0,13 |
|  |  | 0,123 | 0,124 | 0,14 | 0,115 | 0,197 | 0,147 | 0,099 |
|  | + | 1,86 | 1,495 | 1,65 | 1,92 | 1,813 | 1,98 | 1,803 |
|  |  | 0,137 | 0,103 | 0,155 | 0,163 | 0,35 | 0,137 | 0,12 |
|  |  | 1,24 | 1,54 | 1,51 | 1,39 | 1,327 | 1,57 | 1,437 |
|  |  | 0,81 | 0,475 | 0,83 | 2,88 | 0,967 | 0,857 | 0,85 |
| DR15 MS5 |  |  |  |  |  |  |  |  |
|  | - | 0,283 | 0,145 | 0,31 | 0,373 | 0,347 | 0,35 | 0,313 |
|  |  | 0,027 | 0,024 | 0,045 | 0,015 | 0,028 | 0,017 | 0,008 |
|  |  | 0,84 | 0,46 | 0,845 | 0,857 | 0,92 | 0,803 | 0,68 |
|  | + | 0,078 | 0,125 | 0,11 | 0,068 | 0,060 | 0,098 | 0,078 |
|  |  | 0,025 | 0,045 | 0,031 | 0,022 | 0,017 | 0,028 | 0,037 |
|  |  | 0,053 | 0,070 | 0,056 | 0,031 | 0,057 | 0,04 | 0,040 |
|  |  | 0,25 | 0,2 | 0,32 | 0,193 | 0,2 | 0,283 | 0,297 |
