# Supplementary material for: Spontaneous human CD8 T cell and autoimmune encephalomyelitis-induced CD4/CD8 T cell lesions in the brain and spinal cord of HLA-DRB1*15-positive multiple sclerosis humanized immune system mice
Source: eLife. 2024 Jun 20;12:RP88826. doi: 10.7554/eLife.88826 (PMC11189630; doi:10.7554/eLife.88826)
Supplement: Figure 1—figure supplement 5—source data 1. [file elife-88826-fig1-figsupp5-data1.docx]

| DR13 MS | CD4 T cells | | | | |
| --- | --- | --- | --- | --- | --- |
| non-imm | 43,80 | 53,10 | 54,70 |  |  |
| imm 2x200μg | 35,20 | 43,60 | 40,50 | 60,70 | 45,10 |
|  | CD8 T cells | | | | |
| non-imm | 23,00 | 6,88 | 17,50 |  |  |
| imm 2x200μg | 11,00 | 10,30 | 21,80 | 8,46 | 15,90 |
|  | CD19 T cells | | | | |
| non-imm | 27,40 | 34,60 | 23,40 |  |  |
| imm 2x200μg | 47,00 | 41,30 | 30,30 | 23,10 | 34,00 |

**Fig. 1- figure supplement 5- source data 1: Human immune cell engraftment in PBMC humanized B2m-NOG mouse splenocyte analysis**

| DR15 HI | CD4 T cells | | | | |
| --- | --- | --- | --- | --- | --- |
| non-imm | 57,00 | 75,50 | 60,60 |  |  |
| imm 2x200μg | 67,80 | 78,30 | 64,30 | 72,400 | 80,40 |
|  | CD8 T cells | | | | |
| non-imm | 34,30 | 11,00 | 27,90 |  |  |
| imm 2x200μg | 17,20 | 18,70 | 25,20 | 24,30 | 16,80 |
|  | CD19 T cells | | | | |
| non-imm | 2,49 | 2,93 | 2,42 |  |  |
| imm 2x200μg | 2,59 | 1,05 | 9,21 | 2,80 | 0,51 |

| DR15 MS1 | CD4 T cells | | | | |
| --- | --- | --- | --- | --- | --- |
| non-imm | 45,80 | 42,00 | 49,40 |  |  |
| imm 2x200μg | 63,60 | 57,00 | 52,90 | 41,50 | 62,40 |
|  | CD8 T cells | | | | |
| non-imm | 49,80 | 43,70 | 41,30 |  |  |
| imm 2x200μg | 33,10 | 36,90 | 38,90 | 54,30 | 33,30 |
|  | CD19 T cells | | | | |
| non-imm | 0,54 | 2,99 | 1,810 |  |  |
| imm 2x200μg | 0,67 | 2,03 | 1,94 | 1,29 | 2,34 |

| DR15 MS2 | CD4 T cells | | | | |
| --- | --- | --- | --- | --- | --- |
| non-imm | 82,40 | 55,80 | 40,70 |  |  |
| imm 1x100μg | 88,20 | 85,50 | 73,40 | 61,70 |  |
|  | CD8 T cells | | | | |
| non-imm | 9,02 | 39,90 | 48,40 |  |  |
| imm 1x100μg | 8,13 | 7,71 | 12,90 | 33,10 |  |
|  | CD19 T cells | | | | |
| non-imm | 7,21 | 1,12 | 0,077 |  |  |
| imm 1x100μg | 1,71 | 5,75 | 8,78 | 0,36 |  |

| DR15 MS3 | CD4 T cells | | | | |
| --- | --- | --- | --- | --- | --- |
| non-imm | 64,10 | 65,70 | 59,80 |  |  |
| imm 1x100μg | 81,60 | 89,40 | 76,50 | 86,00 |  |
|  | CD8 T cells | | | | |
| non-imm | 11,80 | 29,20 | 12,50 |  |  |
| imm 1x100μg | 10,70 | 4,22 | 15,20 | 9,32 |  |
|  | CD19 T cells | | | | |
| non-imm | 22,20 | 2,37 | 21,10 |  |  |
| imm 1x100μg | 5,44 | 4,71 | 4,36 | 1,49 |  |

| DR15 MS4 | CD4 T cells | | | | |
| --- | --- | --- | --- | --- | --- |
| non-imm | 65,90 | 81,00 | 59,80 |  |  |
| imm 1x100μg | 92,10 | 62,90 | 78,90 | 77,40 |  |
|  | CD8 T cells | | | | |
| non-imm | 26,10 | 13,90 | 29,400 |  |  |
| imm 1x100μg | 6,79 | 27,80 | 16,500 | 15,70 |  |
|  | CD19 T cells | | | | |
| non-imm | 0,39 | 0,30 | 3,07 |  |  |
| imm 1x100μg | 0,40 | 4,49 | 0,36 | 0,20 |  |

| DR15 MS5 | CD4 T cells | | | | |
| --- | --- | --- | --- | --- | --- |
| non-imm | 74,00 | 75,40 | 63,60 |  |  |
| imm 1x100μg | 68,20 | 70,90 | 77,90 | 64,700 |  |
|  | CD8 T cells | | | | |
| non-imm | 16,30 | 15,60 | 30,90 |  |  |
| imm 1x100μg | 25,40 | 18,10 | 14,00 | 32,60 |  |
|  | CD19 T cells | | | | |
| non-imm | 7,87 | 7,55 | 4,72 |  |  |
| imm 1x100μg | 4,38 | 7,74 | 6,99 | 1,41 |  |
