# Supplementary material for: Spontaneous human CD8 T cell and autoimmune encephalomyelitis-induced CD4/CD8 T cell lesions in the brain and spinal cord of HLA-DRB1*15-positive multiple sclerosis humanized immune system mice
Source: eLife. 2024 Jun 20;12:RP88826. doi: 10.7554/eLife.88826 (PMC11189630; doi:10.7554/eLife.88826)
Supplement: Figure 1—figure supplement 6—source data 1. [file elife-88826-fig1-figsupp6-data1.docx]

**Fig. 1- figure supplement 6- source data 1: Comparative flow cytometry analysis of mouse CD11b+ myeloid cell subpopulations in the peripheral blood of non-PBMC-engrafted mouse strains**

| **Mouse strain** | | **%mCD11b^+^** | | **%Ly6C^hi^ of mCD11b^+^** | **%Ly6G^+^ of mCD11b^+^** |
| --- | --- | --- | --- | --- | --- |
| **B2m-NOG** | | | | | |
| Naïve | 94.9 | | 12.7 | | 56.7 |
|  | 98.8 | | 6.48 | | 79.3 |
|  | 95.1 | | 6.41 | | 70.2 |
|  | 96.7 | | 3.19 | | 90.3 |
| **NOD-*scid*** | | | | | |
| Naïve | | 87,6 | | 12,7 | 54,7 |
| CFA dpi 8 | | 86,7 | | 13,1 | 56,6 |
|  |  | 96,9 | | 14,3 | 68,8 |
| **C57BL/6** | | | | | |
| Naïve | | 9.92 | | 34.9 | 12.4 |
|  |  | 11 | | 38 | 13.9 |
| CFA dpi 8 | | 46.5 | | 66.4 | 15.5 |
|  |  | 38.3 | | 61.3 | 17.6 |
|  |  | 31.8 | | 67 | 15.3 |
|  |  | 48.6 | | 69.7 | 13.5 |
|  |  | 37.7 | | 66.8 | 13.2 |

|  |  |  |  |
| --- | --- | --- | --- |
