# Supplementary material for: Spontaneous human CD8 T cell and autoimmune encephalomyelitis-induced CD4/CD8 T cell lesions in the brain and spinal cord of HLA-DRB1*15-positive multiple sclerosis humanized immune system mice
Source: eLife. 2024 Jun 20;12:RP88826. doi: 10.7554/eLife.88826 (PMC11189630; doi:10.7554/eLife.88826)
Supplement: Figure 2—source data 1. [file elife-88826-fig2-data1.docx]

**Fig. 2- source data 1: Human T cells accumulate at brain borders in non-immunized DR15 MS and DR15 HI mice, and form spontaneous parenchymal lesions in brain of DR15 MS mice**

Figure 2D: Borders

|  | hCD8 T+ cells | | | hCD4 T+ cells | | |
| --- | --- | --- | --- | --- | --- | --- |
| DR13 MS | 0,0 | 0,0 | 84,0 |  |  |  |
| DR15 HI | 160,0 | 160,0 | 14,5 | 0,00 | 110,00 | 2,00 |
| DR15 MS1 | 208,0 | 86,5 | 175,0 | 62,50 | 22,00 | 67,00 |
| DR15 MS2 | 24,0 | 16,0 | 1,0 | 0,00 | 52,00 | 30,00 |
| DR15 MS3 | 120,0 | 90,0 | 158,0 | 14,00 | 0,00 | 45,00 |
| DR15 MS4 | 0,0 | 6,0 | 11,0 | 0,00 | 0,00 | 0,00 |
| DR15 MS5 | 66,0 | 185,0 | 218,0 | 0,00 | 178,00 | 82,00 |

Figure 2D: Parenchyma

|  | hCD8+ T cells | | | hCD4+ T cells | | |
| --- | --- | --- | --- | --- | --- | --- |
| DR13 MS | 0,000000 | 0,000000 | 0,000000 | 0,000000 | 0,000000 | 0,000000 |
| DR15 HI | 0,5595897 | 2,374188 | 0,1766624 | 0,000000 | 0,1104274 | 0,000000 |
| DR15 MS1 | 3,308269 | 0,9259421 | 3,340046 | 0,4766151 | 0,1821525 | 0,5444127 |
| DR15 MS2 | 0,2761329 | 0,1139161 | 0,000000 | 0,000000 | 0,000000 | 0,1260023 |
| DR15 MS3 | 1,592610 | 1,211872 | 1,597852 | 0,000000 | 0,000000 | 0,1205926 |
| DR15 MS4 | 0,000000 | 0,000000 | 0,000000 | 0,000000 | 0,000000 | 0,000000 |
| DR15 MS5 | 1,842911 | 4,274342 | 9,697695 | 0,000000 | 3,894400 | 0,2131362 |

Figure 2E: CD4/CD8 ratio

|  | barriers | parenchyma |
| --- | --- | --- |
| DR15 HI | 0,334828 | 0,0349345 |
| DR15 MS1 | 0,322684 | 0,1583969 |
| DR15 MS3 | 0,1603261 | 0,02857143 |
| DR15 MS5 | 0,554371 | 0,2774194 |
