# Supplementary material for: Spontaneous human CD8 T cell and autoimmune encephalomyelitis-induced CD4/CD8 T cell lesions in the brain and spinal cord of HLA-DRB1*15-positive multiple sclerosis humanized immune system mice
Source: eLife. 2024 Jun 20;12:RP88826. doi: 10.7554/eLife.88826 (PMC11189630; doi:10.7554/eLife.88826)
Supplement: Figure 2—figure supplement 1—source data 1. [file elife-88826-fig2-figsupp1-data1.docx]

**Fig. 2- figure supplement 1- source data 1: Inflammation in peripheral GVHD target tissues lung (A) and liver (B) in PBMC B2m-NOG mice.**

Liver inflammation score (immunized mice)

| DR13 MS | DR15 HI | DR15 MS1 |
| --- | --- | --- |
| 0,4833333 | 2,133333 | 1,083333 |
| 0,1166667 | 1,850000 | 1,600000 |
| 0,3166667 | 2,300000 | 1,216667 |
| 0,4166667 | 2,200000 | 0,450000 |
|  | 2,633333 | 1,000000 |

Liver inflammation score (non immunized mice)

| DR13 MS | DR15 HI | DR15 MS |
| --- | --- | --- |
| 1,533333 | 3,050000 | 0,5333334 |
| 1,800000 | 1,750000 | 2,400000 |
| 1,383333 | 0,7666667 | 1,800000 |

Lung inflammation score (immunized mice)

| DR13 MS | DR15 HI | DR15 MS |
| --- | --- | --- |
| 0,50 | 3,00 | 2,25 |
| 1,25 | 2,00 | 1,85 |
| 0,35 | 2,15 | 1,25 |
| 0,50 | 2,40 | 1,50 |
| 0,60 | 2,50 | 2,25 |
|  |  |  |

Lung inflammation score (non-immunized mice)

| DR13 MS | DR15 HI | DR15 MS |
| --- | --- | --- |
| 2,25 | 2,25 | 1,75 |
| 2,00 | 3,00 | 2,00 |
|  | 1,25 | 2,15 |
