# Supplementary material for: Spontaneous human CD8 T cell and autoimmune encephalomyelitis-induced CD4/CD8 T cell lesions in the brain and spinal cord of HLA-DRB1*15-positive multiple sclerosis humanized immune system mice
Source: eLife. 2024 Jun 20;12:RP88826. doi: 10.7554/eLife.88826 (PMC11189630; doi:10.7554/eLife.88826)
Supplement: Figure 3—source data 1. [file elife-88826-fig3-data1.docx]

**Fig. 3- source data 1: Human T cells infiltrate spinal cord white matter in non-immunized DR15 MS and DR15 HI mice, and form grey matter lesions in DR15 MS mice**

Figure 3B: Parenchymal CD3 score

| DR15 HI | DR15 MS1 |
| --- | --- |
| 0,375 | 3,250000 |
| 2,375 | 3,250000 |
| 1,000 | 2,166667 |

Figure 3Ci: hCD3 G.M. lesions/spinal cord section

| DR15 HI | DR15 MS |
| --- | --- |
| 0,0 | 1,5000 |
| 0,0 | 1,0000 |
| 0,5 | 1,3333 |

Figure 3Cii: hCD3 W.M. lesions/spinal cord section

| DR15 HI | DR15 MS |
| --- | --- |
| 0, | 1, |
| 3, | 1, |
| 0, | 0, |

Figure 3D: CD4/CD8 ratio

| DR15 HI  BORDERS | DR15 HI PARENCHYMA | DR15 MS1 BORDERS | DR15 MS1 PARENCYMA |
| --- | --- | --- | --- |
| 0,1282051 | 0,01315789 | 0,2645161 | 0,2166667 |
