# Supplementary material for: Spontaneous human CD8 T cell and autoimmune encephalomyelitis-induced CD4/CD8 T cell lesions in the brain and spinal cord of HLA-DRB1*15-positive multiple sclerosis humanized immune system mice
Source: eLife. 2024 Jun 20;12:RP88826. doi: 10.7554/eLife.88826 (PMC11189630; doi:10.7554/eLife.88826)
Supplement: Figure 4—source data 1. [file elife-88826-fig4-data1.docx]

|  |  |  |  |  |
| --- | --- | --- | --- | --- |

**Fig. 4- source data 1: Immunization with myelin peptides increases hCD4 T cell infiltration of brain parenchyma resulting in mixed hCD4/hCD8 T cell lesions in brain of DR15 MS and DR15 HI mice**

Figure 4C: Borders

|  | hCD45 cells/ whole brain section  non-immunized borders | | | hCD45 cells/ whole brain section  immunized borders | | | | |
| --- | --- | --- | --- | --- | --- | --- | --- | --- |
| DR15 HI | 200, | 460, | 4, | 453, | 65, | 180, | 397, | 40, |
| DR15 MS1 | 344, | 111, | 278, | 530, | 1010, | 208, | 517, | 78, |

Figure 4C: Parenchyma

|  | hCD45 cells/mm2  non immunized parenchyma | | | hCD45 cells/mm2  immunized parenchyma | | | | |
| --- | --- | --- | --- | --- | --- | --- | --- | --- |
| DR15 HI | 0,7630768 | 3,091966 | 0,000000 | 3,951078 | 0,000000 | 4,155846 | 3,403840 | 0,03341883 |
| DR15 MS1 | 3,308269 | 0,1821525 | 2,148223 | 3,971326 | 6,313768 | 0,4160131 | 13,783140 | 0,000000 |

Figure 4 D: Borders

|  | hCD8+ T cells/ whole brain section  non immunized | | | | | hCD8+ T cells/ whole brain section  immunized | | | | |
| --- | --- | --- | --- | --- | --- | --- | --- | --- | --- | --- |
| DR13 MS | 0,0 | 0,0 | 84,0 |  |  | 1,0 | 2,0 | 0,0 | 0,0 | 1,0 |
| DR15 HI | 160,0 | 160,0 | 14,5 |  |  | 118,5 | 28,0 | 74,5 | 144,0 | 103,5 |
| DR15 MS1 | 208,0 | 86,5 | 175,0 |  |  | 165,5 | 374,0 | 0,0 | 233,0 | 18,0 |
| DR15 MS2 | 24,0 | 16,0 | 1,0 |  |  | 0,0 | 0,0 | 18,0 | 6,0 |  |
| DR15 MS3 | 120,0 | 90,0 | 158,0 |  |  | 8,0 | 10,0 | 150,0 | 108,0 |  |
| DR15 MS4 | 0,0 | 6,0 | 11,0 |  |  | 4,0 | 0,0 | 4,0 | 4,0 |  |
| DR15 MS5 | 66,0 | 185,0 | 218,0 |  |  | 12,0 | 5,0 | 155,0 | 0,0 |  |

Figure 4D: Borders

|  | hCD4+ T cells/ whole brain section  non immunized | | | | | hCD4+ T cells/ whole brain section  immunized | | | | |
| --- | --- | --- | --- | --- | --- | --- | --- | --- | --- | --- |
|  |  |  |  |  |  |  |  |  |  |  |
| DR15 HI | 0,00 | 110,00 | 2,00 |  |  | 280, | 32, | 67, | 241, | 9, |
| DR15 MS1 | 62,50 | 22,00 | 67,00 |  |  | 258, | 347,5 | 0, | 276,5 | 130, |
| DR15 MS2 | 0,00 | 52,00 | 30,00 |  |  | 46, | 0, | 124, | 431, |  |
| DR15 MS3 | 14,00 | 0,00 | 45,00 |  |  | 58, | 348, | 408, | 154, |  |
| DR15 MS4 | 0,00 | 0,00 | 0,00 |  |  | 50, | 0, | 0, | 0, |  |
| DR15 MS5 | 0,00 | 178,00 | 82,00 |  |  | 0, | 0, | 117, | 0, |  |

Figure 4D: Parenchyma

Figure 4D: Parenchyma

|  | hCD8+ T cells/mm2  non immunized | | | | | hCD8+ T cells/mm2  immunized | | | | |  |
| --- | --- | --- | --- | --- | --- | --- | --- | --- | --- | --- | --- |
| DR13 MS | 0,000000 | 0,000000 | 0,000000 |  |  | 0,000000 | 0,000000 | 0,000000 | 0,000000 | 0,000000 |  |
| DR15 HI | 0,5595897 | 2,374188 | 0,1766624 |  |  | 0,8903838 | 0,300952 | 2,689077 | 1,916448 | 1,687651 |  |
| DR15 MS1 | 3,308269 | 0,9259421 | 3,340046 |  |  | 1,311287 | 7,384854 | 0,000000 | 8,426307 | 0,05756947 |  |
| DR15 MS2 | 0,2761329 | 0,1139161 | 0,000000 |  |  | 0,000000 | 0,000000 | 0,2386241 | 0,1764874 |  |  |
| DR15 MS3 | 1,592610 | 1,211872 | 1,597852 |  |  | 0,000000 | 0,02762521 | 3,993310 | 1,456289 |  |  |
| DR15 MS4 | 0,000000 | 0,000000 | 0,000000 |  |  | 0,000000 | 0,000000 | 0,000000 | 0,000000 |  |  |
| DR15 MS5 | 1,842911 | 4,274342 | 9,697695 |  |  | 0,000000 | 0,000000 | 2,747222 | 0,000000 |  |  |

|  | hCD4+ T cells/mm2  non immunized | | |  | hCD4+ T cells/mm2  immunized | | | | |
| --- | --- | --- | --- | --- | --- | --- | --- | --- | --- |
| DR13 MS | 0,000000 | 0,000000 | 0,000000 |  | 0,000000 | 0,000000 | 0,000000 | 0,000000 | 0,000000 |
| DR15 HI | 0,000000 | 0,1104274 | 0,000000 |  | 1,099067 | 0,000000 | 1,436211 | 1,029733 | 0,2673507 |
| DR15 MS1 | 0,4766151 | 0,1821525 | 0,5444127 |  | 1,273822 | 2,381756 | 0,000000 | 3,172778 | 0,4605558 |
| DR15 MS2 | 0,000000 | 0,000000 | 0,1260023 |  | 0,1579966 | 0,000000 | 0,2982802 | 5,265208 |  |
| DR15 MS3 | 0,000000 | 0,000000 | 0,1205926 |  | 0,2507549 | 3,204524 | 7,266516 | 1,985849 |  |
| DR15 MS4 | 0,000000 | 0,000000 | 0,000000 |  | 0,1724209 | 0,000000 | 0,000000 | 0,000000 |  |
| DR15 MS5 | 0,000000 | 3,894400 | 0,2131362 |  | 0,000000 | 0,000000 | 0,1962301 | 0,000000 |  |

Figure 4E: Borders CD4/CD8 ratio

|  | non immunized | immunized |
| --- | --- | --- |
| DR15 HI | 0,334828 | 1,342583 |
| DR15 MS1 | 0,322684 | 1,280202 |
| DR15 MS3 | 0,1603261 | 3,507246 |
| DR15 MS5 | 0,554371 | 0,6802326 |

Figure 4E: Parenchyma CD4/CD8 ratio

|  | non immunized | immunized |
| --- | --- | --- |
| DR15 HI | 0,0349345 | 0,5272727 |
| DR15 MS1 | 0,1583969 | 0,4302326 |
| DR15 MS3 | 0,02857143 | 2,431138 |
| DR15 MS5 | 0,2774194 | 0,07142857 |

Figure 4L

| CD45 lesions | hCD**4 T cells** |
| --- | --- |
| 0, | 0,157997 |
| 0, | 0,000000 |
| 0, | 0,298280 |
| 1, | 5,265208 |
| 1, | 0,250755 |
| 0, | 3,204524 |
| 4, | 7,266516 |
| 1, | 1,985849 |
| 0, | 0,172421 |
| 0, | 0,000000 |
| 0, | 0,000000 |
| 1, | 0,000000 |
| 1, | 0,000000 |
| 0, | 0,000000 |
| 1, | 0,196230 |
| 0, | 0,000000 |
| 0, | 1,099067 |
| 0, | 0,000000 |
| 1, | 1,436211 |
| 0, | 1,029733 |
| 0, | 0,267351 |
| 1, | 1,273822 |
| 2, | 2,381756 |
| 0, | 0,000000 |
| 3, | 3,172778 |
| 0, | 0,460556 |

| CD45 lesions | hCD**8 T cells** |
| --- | --- |
| 0, | 0,000000 |
| 0, | 0,000000 |
| 0, | 0,238624 |
| 1, | 0,176487 |
| 1, | 0,000000 |
| 0, | 0,027625 |
| 4, | 3,993310 |
| 1, | 1,456289 |
| 0, | 0,000000 |
| 0, | 0,000000 |
| 0, | 0,000000 |
| 1, | 0,000000 |
| 1, | 0,000000 |
| 0, | 0,000000 |
| 1, | 2,747222 |
| 0, | 0,000000 |
| 0, | 0,890384 |
| 0, | 0,300952 |
| 1, | 2,689077 |
| 0, | 1,916448 |
| 0, | 1,687651 |
| 1, | 1,311287 |
| 2, | 7,384854 |
| 0, | 0,000000 |
| 3, | 8,426307 |
| 0, | 0,057569 |
