# Supplementary material for: Spontaneous human CD8 T cell and autoimmune encephalomyelitis-induced CD4/CD8 T cell lesions in the brain and spinal cord of HLA-DRB1*15-positive multiple sclerosis humanized immune system mice
Source: eLife. 2024 Jun 20;12:RP88826. doi: 10.7554/eLife.88826 (PMC11189630; doi:10.7554/eLife.88826)
Supplement: Figure 5—source data 1. [file elife-88826-fig5-data1.docx]

**Fig. 5- source data 1: Immunization with myelin peptides increases hCD4 T cell infiltration of spinal cord white matter in both DR15 MS and DR15 HI mice**

Figure 5B: Parenchymal CD3 score

| DR15 HI | DR15 MS1 |
| --- | --- |
| 2,000000 | 3,125000 |
| 1,250000 | 1,375000 |
| 2,125000 | 1,666667 |
| 1,833333 | 3,125000 |
| 1,750000 | 2,000000 |

Figure 5Ci: hCD3 T cells in grey matter (GM) lesions/spinal cord section

| DR15 HI | DR15 MS1 |
| --- | --- |
| 0,0 | 0, |
| 0,0 | 0, |
| 0,0 | 0, |
| 0,0 | 0, |
| 0,5 | 0, |

Figure 5Cii: hCD3 T cells in white matter (WM) lesions/spinal cord section

| DR15 HI | DR15 MS |
| --- | --- |
| 0,000000 | 0,0 |
| 0,500000 | 0,5 |
| 1,000000 | 0,0 |
| 0,666667 | 1,0 |
| 0,000000 | 1,5 |

Figure 5Di: CD4/CD8 ratio borders

| DR15 HI  Non-immunized | DR15 HI  Immunized | DR15 MS1  Non-immunized | DR15 MS  Immunized |
| --- | --- | --- | --- |
| 0,1282051 | 0,8032787 | 0,2645161 | 0,953271 |

Figure 5Dii: CD4/CD8 ratio parenchyma

| DR15HI  Non-immunized | DR15  Immunized | DR15MS1  Non-immunized | DR15MS  Immunized |
| --- | --- | --- | --- |
| 0,01315789 | 0,3333333 | 0,2166667 | 0,4634146 |
